# The relationship between cardiometabolic index and infertility in American adults: a population-based study

**DOI:** 10.3389/fendo.2024.1424033

**Published:** 2024-09-04

**Authors:** Huifang Cheng, Xiaoli He, Xiaoke Jin

**Affiliations:** ^1^ Department of Obstetrics and Gynecology, Lishui Hospital of Traditional Chinese Medicine, Lishui, Zhejiang, China; ^2^ Department of Obstetrics and Gynecology, Affiliated Hospital of Jining Medical College, Jining, Shandong, China

**Keywords:** cross-sectional study, NHANES, infertility, cardiometabolic index, IR

## Abstract

**Background:**

Infertility is a fertility disorder caused by various factors, with lipid metabolism playing a crucial role in its development. The cardiometabolic index (CMI), which combines blood lipids (TG/HDL-C) and obesity-related parameters (WHtR), is a new quantitative indicator. This study used NHANES data to investigate the relationship between CMI and the incidence of infertility.

**Methods:**

We utilized data from women who took part in the National Health and Nutrition Examination Survey (NHANES) from 2015 to March 2020 to calculate the CMI index. Subsequently, we used multivariate logistic regression, smooth curve fitting, and subgroup analysis to investigate the relationship between the CMI index and infertility.

**Results:**

The logistic regression model revealed a significant positive correlation between the CMI index and infertility, even after adjusting for all confounding variables (OR=3.23, 95%CI: 1.55-6.73, p=0.0017). This association remained consistent across all subgroups (p>0.05 for all interactions). Smooth curve fitting demonstrated a positive nonlinear relationship between CMI and infertility. However, the CMI index had limited diagnostic power for infertility (AUC=0.60, 95%CI: 0.56-0.65). However, the CMI-BMI index combined with BMI had good predictive performance (AUC=0.722, 95%CI: 0.676-0.767).

**Conclusion:**

The CMI index shows a positive correlation with infertility, but its diagnostic value is restricted. The combination with BMI has good diagnostic value. Further investigation is required to determine the effectiveness of the CMI index as an early indicator of infertility.

## 1 Introduction

Infertility is defined as the inability to become pregnant after engaging in regular, unprotected sexual intercourse for over a year without using any form of contraception (1, 2). Around 7% to 15.5% of women in the United States who are of childbearing age experience infertility (3). Infertility is considered a significant public health issue by the World Health Organization (WHO), impacting approximately 186 million individuals globally. The condition affects up to 15% of women in their childbearing years (4–6). It is estimated that over half of women who are unable to conceive suffer from severe mental health conditions like depression, anxiety, and social dysregulation.

Various epidemiological studies have revealed that infertility is a fertility disorder with multiple causes. Previous studies have shown that factors such as obesity, alcohol consumption, smoking, education level, and past medical history (1, 7–9) are associated with female infertility. Another retrospective study highlighted that smoking was associated with increased PSA levels in men undergoing radical prostatectomy (10). Therefore, the role of lifestyle interventions may not be limited to the management of metabolic markers, but may be critical in the prevention and management of chronic diseases more broadly. Recent research has shown that female infertility caused by polycystic ovary syndrome, obesity, thyroid dysfunction, and endometriosis is linked to a higher risk of cardiovascular disease (CVD) (11). Women with infertility have a higher prevalence of cardiovascular risk factors even before pregnancy (12), suggesting that common risk factors such as diabetes, chronic hypertension (13) and obesity (14) may underlie infertility and cardiovascular disease. Cardiovascular disease (CVD) has become the leading cause of death worldwide, and although it can be effectively treated, there are still serious complications that seriously affect the quality of life (15). Research has indicated a strong correlation between female infertility and metabolic syndrome, as well as its individual components like dyslipidemia and hypertension, in the later stages of life (16). Considering the correlation between Mets, CVD, and infertility, we hypothesize that there could be a connection between CMI and infertility.

The cardiometabolic index is a novel measure that combines triglycerides, high-density lipoprotein cholesterol, and waist-to-height ratio. It serves as an indicator of visceral adipose tissue distribution and dysfunction, providing insight into an individual’s blood lipid levels and degree of obesity (17–19). The TG/HDL-C ratio has been proven to be a reliable risk factor for cardiovascular disease for a long time (20). In addition, studies have reported that TG/HDL-C ratios are strongly associated with insulin resistance (21) and Mets (22). It is a commonly known fact that waist circumference (WC) is a simple indicator of abdominal obesity, while waist-to-height ratio (WHtR) is considered to be more accurate than waist circumference (23). WHtR has been proven to be a more effective indicator of cardiovascular disease risk factors and infertility when compared to body mass index (BMI) and waist circumference (WC) (24, 25). At present, the link between lipid levels and infertility remains uncertain. Therefore, we analyzed data from the National Health and Nutrition Survey (NHANES) in order to investigate the association between CMI and the prevalence of infertility.

## 2 Materials and methods

### 2.1 Data source

The data for this study were obtained from the National Health and Nutrition Survey (NHANES), a program conducted by the National Center for Health Statistics (NCHS) to evaluate nutrition and health in the United States. The survey utilized a complex multi-stage probabilistic design to ensure a nationally representative sample of non-institutionalized Americans. Participants underwent a family interview to provide information on their health, socioeconomic status, and other relevant factors. Physical and laboratory examinations were then conducted in a mobile examination facility.

Approval for all NHANES study methods was granted by the NCHS Research Ethics Review Committee, with written informed consent obtained from all survey respondents. The CDC website (www.cdc.gov/nchs/nhanes/) provides access to detailed NHANES study design and data for the public. This cross-sectional study followed the criteria for enhanced epidemiological observational reporting (24).

### 2.2 Study population

**Figure 1:**
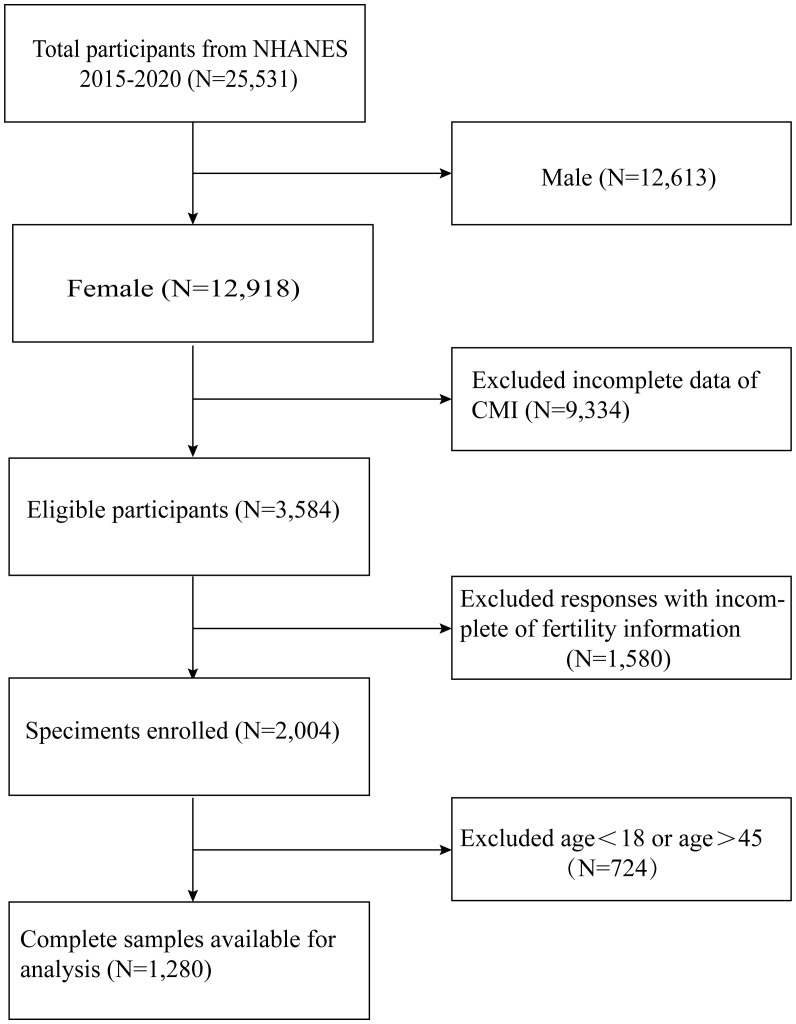
Flow chart for the inclusion and exclusion of study participants.

Health problems related to infertility were only included in the NHANES cycle from 2015 to 2020; Therefore, we use these periods as our data. In our analysis, we included participants with comprehensive information on infertility and CMI. Initially, a total of 25,531 participants participated. After excluding male participants (n=12,613), participants lacking CMI data (n=9,334), fertility information (n=1,580), and women older than 45 years and younger than 18 years (n=724), our final analysis included 1,280 eligible participants (**Figure 1**).

### 2.3 Cardiometabolic index

CMI is calculated using anthropometric indicators obtained from blood samples. Rigorous testing and measurement protocols were followed to ensure the accuracy and consistency of the data. Blood samples were collected either on a research vehicle or at a specific location, and then analyzed in a laboratory. Height and waist circumference of participants were measured by trained health professionals at a mobile screening unit. Using these indicators, WHtR and CMI were calculated individually.


(1) WHtR=waist circumference (cm)/height (cm)

(2) CMI=TG (mmol/L)/HDL−C (mmol/L) ×WHtR.

### 2.4 Infertility

The reproductive health questionnaire for each woman was self-reported as a dependent variable for infertility (name of variable in questionnaire: RHQ 074). The researchers surveyed the subjects, asking questions such as “Trying to conceive for a year?” (26). If the answer is “yes”, it means “sterile”; If the answer is not “yes”, it means “fertile”.

### 2.5 Covariables

Covariates such as sex, age, ethnicity, education level, income-to-poverty ratio (PIR), body mass index (BMI), alcohol use, smoking status, hypertension status, diabetes status, age at menarche, previous treatment for pelvic infection/pelvic inflammatory disease, regular menstruation within 12 months, HDL-C, TC, TG, and LDL-C were all included in the analysis.

### 2.6 Statistical analysis

The statistical analyses for this study were carried out in accordance with Centers for Disease Control and Prevention (CDC) guidelines. When we divided the 2-year weights for each cycle by 2, we obtained the new sample weights for the combined survey periods.

In this study, we compared two groups based on infertility status using weighted Student t-tests for continuous variables and weighted chi-square tests for categorical data. Categorical data were presented as proportions, while continuous variables were described with mean and standard deviation. We used weighted multiple linear and logistic regression to analyze the linear relationship between CMI and infertility. By categorizing CMI into thirds, we conducted a trend test to assess the linear association with infertility. Subgroup analysis was performed to explore how factors such as age, BMI, hypertension, diabetes, menstrual regularity, and menarche age influenced the relationship between CMI and infertility, with interaction tests to assess consistency across subgroups. We also examined the nonlinear relationship between CMI and infertility using smoothing curve fitting. The diagnostic accuracy of the CMI index for infertility was assessed using the ROC curve and calculating the AUC. Statistical analysis was carried out using R Studio (version 4.3.2), with significance set at p<0.05. A weighting strategy was implemented to minimize dataset fluctuations.

## 3 Results

### 3.1 Baseline characteristics

A total of 1,280 participants between the ages of 18 and 45 were included in the study, with 132 of them being infertility patients. The characteristics of the study participants are detailed in **Table 1** based on their infertility status. Older women with a higher BMI, alcohol intake, high blood pressure, and diabetes were found to have a higher prevalence of self-reported infertility. Additionally, women with a higher CMI, averaging 0.54 ± 0.41, also reported higher rates of infertility. At the same time, women with higher triglycerides (TG) also have a higher prevalence of infertility.

**Table 1:** Weighted characteristics of the study population based on infertility.

Characteristic	Control	Infertility	*P*-value
	N=1148	N=132	
Age, (years)	31.17 ± 8.17	33.83 ± 7.18	<0.001
Race (%)			0.701
Mexican American	15.94	16.67	
Other Hispanic	10.54	7.58	
Non-Hispanic white	28.66	33.33	
Non-Hispanic black	25.78	23.48	
Other Races	19.08	18.94	
Education level (%)			0.727
Less than high school	14.96	13.18	
High school or GED	19.56	17.83	
Above high school	65.48	68.99	
Income to poverty ratio	2.28 ± 1.58	2.41 ± 1.60	0.386
BMI (kg/m^2^)			<0.001
<25	35.28	24.24	
25–29.9	23.69	16.67	
≥30	41.03	59.09	
Smoking status (%)			0.935
Every day	44.48	47.06	
Some days	11.71	11.76	
Never	43.81	41.18	
Alcohol drinking status (%)			0.007
Yes	6.77	13.79	
No	93.23	86.21	
Hypertension (%)			0.004
Yes	13.41	22.73	
No	86.59	77.27	
Diabetes (%)			<0.001
Yes	3.75	10.61	
No	96.25	89.39	
Age when first menstrual period occurred (%)			0.240
Age<10	5.49	6.06	
10≤Age<15	81.88	86.36	
15≤Age ≤ 20	12.63	7.58	
Had regular periods in past 12 months (%)			0.783
Yes	89.37	90.15	
No	10.63	9.85	
Ever treated for a pelvic infection/PID (%)			0.265
Yes	4.62	6.82	
No	95.38	93.18	
LDL-C(mmol/L, mean±SD)	2.64 ± 0.77	2.68 ± 0.82	0.544
HDL-C(mmol/L, mean±SD) TC (mmol/L, mean ± SD)	1.47 ± 0.40 4.54 ± 0.92	1.37 ± 0.39 4.55 ± 0.97	0.001 0.899
TG (mmol/L, mean ± SD)	0.96 ± 0.63	0.99 ± 0.49	0.017
WC,cm	95.29 ± 19.22	103.73 ± 20.34	<0.001
WHtR	0.59 ± 0.12	0.64 ± 0.12	<0.001
CMI	0.47 ± 0.46	0.54 ± 0.41	<0.001

Mean ± SD for continuous variables: P-value was calculated by weighted linear regression model.

(%) for categorical variables: P-value was calculated by weighted chi-square test.

BMI, body mass index; TG, triglyceride; HDL-C, high-density lipoprotein cholesterol; LDL-c low-density lipoprotein cholesterol; TC, total cholesterol; WC, waist circumference; CMI cardiometabolic index; PIR poverty income ratio.

### 3.2 Association between CMI and infertility

**Figure 2:**
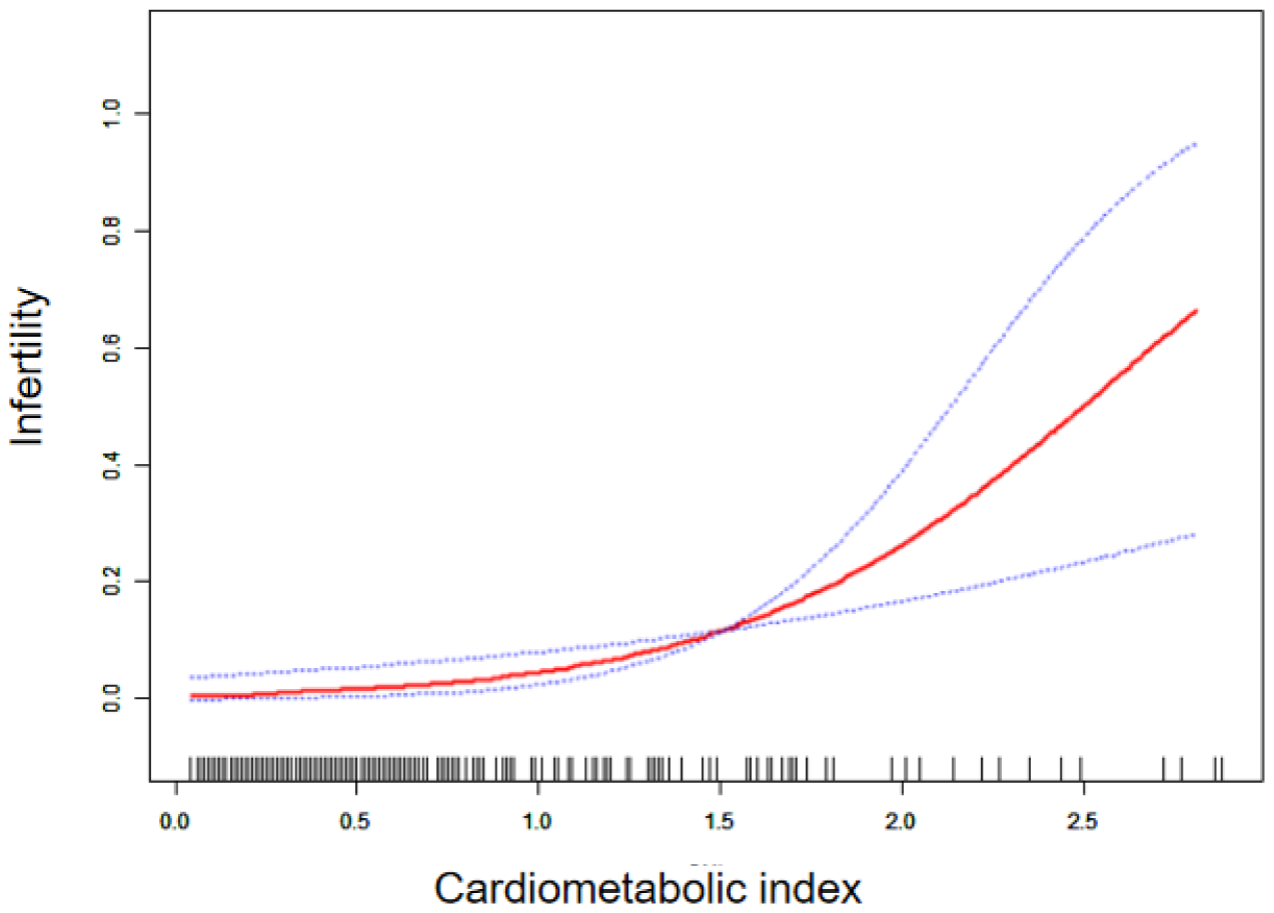
Smoothing curve fitting of CMI index to infertility.

**Table 2** displays the association between CMI and infertility. The findings revealed that as CMI levels increased, the risk of infertility also increased (OR=3.23, 95%CI:1.55, 6.7, p=0.0017) after adjusting for all variables. Even after categorizing CMI into tritiles, the correlation remained statistically significant. Individuals in the highest CMI quartile had a higher risk of infertility compared to those in the lowest CMI tritile (OR=3.68, 95%CI:1.66-8.17). Furthermore, the results from the smooth-fitting curve supported a linear positive relationship between CMI and infertility (see **Figure 2**).

**Table 2:** Association between cardiometabolic index and infertility.

Parameters	Model 1 OR (95%CI), P-value	Model 2 OR (95%CI), P-value	Model 3 OR (95%CI), P-value
CMI index	1.46 (1.17, 1.81) <0.001	1.36 (1.08, 1.70) 0.009	3.23 (1.55, 6.73) 0.0017
CMI index Tertile			
Tertile 1	1(ref)	1(ref)	1(ref)
Tertile 2	2.10 (1.25, 3.53) 0.005	2.04 (1.21, 3.44) 0.0074	2.43 (1.34, 4.42) 0.003
Tertile 3	2.80 (1.70, 4.60) <0.001	2.48 (1.48, 4.14) <0.001	3.68 (1.66, 8.17) 0.001

Model 1: no covariates were adjusted.

Model 2: age and race were adjusted.

Model 3: age,race, education level, BMI, smoking status, alcohol status, diabetes status, hypertension status, Age when first menstrual period occurred, Had regular periods in past 12 months, Ever treated for a pelvic infection/PID, and PIR were adjusted.

95%CI, 95 % confidence interval.

### 3.3 Subgroup analyses

We conducted subgroup analyses to determine if the association between CMI and infertility remained consistent across different factors. Our results indicate that there is no link between CMI and infertility. As demonstrated in **Table 3**, variables such as age, BMI, hypertension, diabetes, age at menarche, and menstrual regularity over the past year did not have a significant impact on the positive relationship between CMI and infertility (all p>0.05).

**Table 3:** Subgroups analyses of the effect of CMI on infertility.

Subgroup	Infertility [OR(95%CI)]	P for interaction
Age		0.0845
Tertile1	2.29 (0.75, 7.01)	
Tertile2	1.55 (0.74, 3.26)	
Tertile3	0.91 (0.49, 1.71)	
BMI		0.4391
<25	1.76 (0.99, 3.11)	
25-29.9	0.99 (0.55, 1.77)	
≥30	1.23 (0.88, 1.74)	
Hypertension		0.5694
Yes	1.00 (0.28, 3.60)	
No	1.50 (0.84, 2.67)	
Diabetes		0.8691
Yes	1.41 (0.26, 7.68)	
No	1.24 (0.71, 2.14)	
Had regular periods in past 12 months		0.6091
Yes	0.97 (0.53, 1.76)	
No	1.00 (0.39, 2.59)	
Age when first menstrual period occurred		0.8291
Age<10	1.18 (0.24, 5.94)	
10≤Age<15	1.33 (0.77, 2.30)	
15≤Age≤20	0.96 (0.21, 4.45)	

### 3.4 Diagnostic efficacy of CMI index for infertility

**Figure 3:**
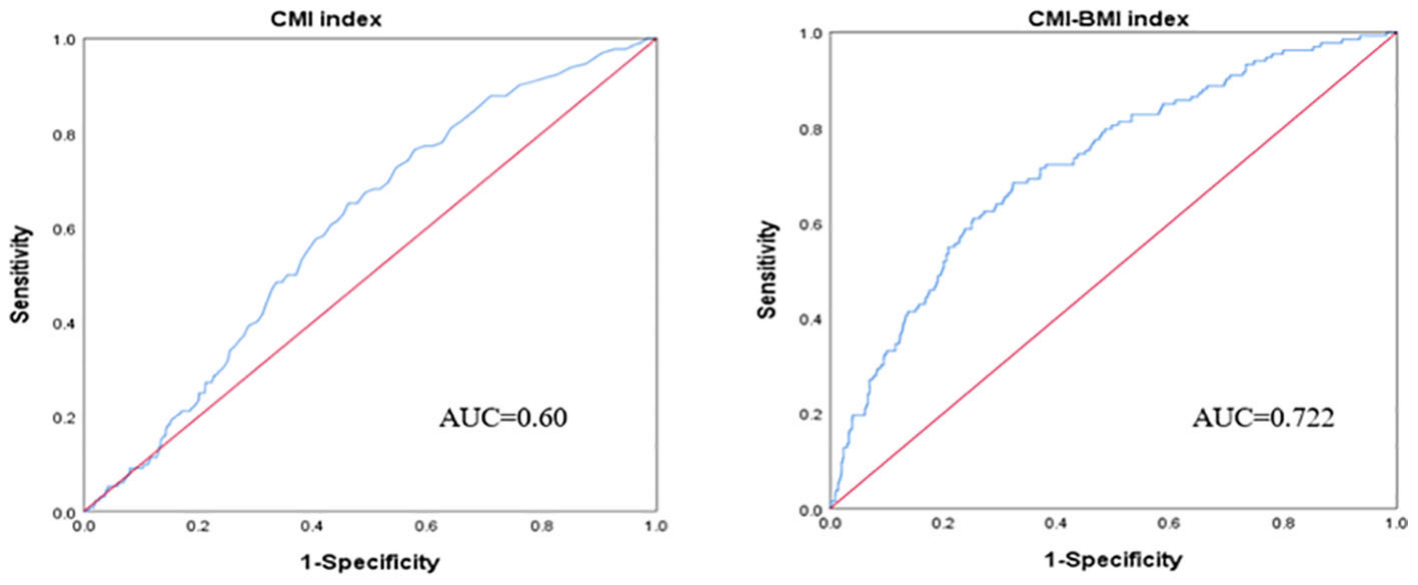
ROC curve of the CMI index and CMI-BMI index used to diagnose infertility.

The diagnostic validity of the CMI index was assessed through the receiver operating characteristic (ROC) curve (**Figure 3**), revealing a cut-off value of 0.345 for diagnosing infertility. The AUC was 0.60 (95%CI: 0.56-0.65) with a sensitivity of 65.2% and specificity of 53.8%. However, we found that CMI in combination with BMI can improve its diagnostic utility. The cut-off value of CMI-BMI index was 12.652, the AUC was 0.722 (95%CI: 0.676-0.767), the sensitivity was 68.4%, and the specificity was 67.7%.

## 4 Discussion

The study examined the connection between CMI levels and infertility using data from the NHANES database. It found that CMI levels were higher in the infertility group compared to the non-infertility group, and that infertility risk increased with higher CMI levels. Smooth curve fitting analysis revealed a positive linear relationship between CMI and infertility. Even after adjusting for various factors, there was still a significant correlation between CMI and infertility, suggesting that CMI could be a useful indicator for assessing infertility in the future. However, the diagnostic accuracy of the CMI index for infertility is limited, and more research is needed to fully explore its potential as an early predictor of infertility risk. However, the combination of CMI and BMI is more useful in diagnosing infertility, probably because CMI-BMI contains not only blood lipids and cardiometabolic abnormalities compared to the CMI index, but also the BMI, one of the obesity indexes, to improve its diagnostic ability.

This study is believed to be the first to examine the connection between CMI and infertility. Wakabayashi et al. initially introduced CMI, which combines indicators of blood lipids and obesity (TG/HDL-C, WHtR) in a novel way, demonstrating that CMI is a valuable new tool for detecting diabetes mellitus (18). Yin et al. discovered that abdominal obesity, which is defined by the presence of visceral fat, is closely linked to metabolic disorders. Their analysis of 1509 female adults in the United States revealed a strong correlation between waist circumference and/or abdominal obesity and a heightened risk of infertility (27). Both infertility and obesity are often associated with high serum lipid concentrations and increased cortisol levels. It is worth noting that most obese individuals tend to have consistently elevated blood lipid levels, which can create a harmful cycle (28). In this study, it was found that having a BMI of 30kg/m2 or higher can increase the risk of infertility. This may be due to the fact that BMI is closely associated with obesity-related metabolic disorders, leading to the accumulation of visceral fat and an increased risk of cardiovascular disease. A randomized trial involving women aged 18 to 40 years also showed that elevated levels of triglycerides, total cholesterol, and LDL cholesterol, as well as decreased levels of HDL cholesterol, were linked to a higher risk of female infertility (29). Cai et al. carried out a randomized controlled trial involving 1000 patients diagnosed with PCOS. They examined the impact of preconception LDLC, HDL-C, and TG levels on pregnancy outcomes and discovered a negative correlation between serum lipid concentration and reproductive results. Additionally, they determined that high LDL-C levels were linked to ovulation, clinical pregnancy, and a decreased likelihood of live birth (30). Our study revealed that elevated levels of triglycerides (TG) were linked to female infertility, while the impact of low-density lipoprotein cholesterol (LDL-C) on female infertility was not significant. Furthermore, infertility was strongly correlated with individuals who had hypertension or diabetes. These findings suggest that infertility may be influenced by lipid metabolism disorders, particularly in individuals with hypertension and diabetes. Additionally, infertility could potentially disrupt lipid metabolism and increase the risk of cardiovascular diseases in affected individuals. In addition to the above systemic metabolic factors, local reproductive pathology also plays a key role in infertility. One study highlighted the prevalence of varicocele in men with primary infertility by up to 35% (19), suggesting that we can propose a multifaceted approach to infertility treatment and research by exploring the local link between these systemic metabolic disorders and varicocele.

CMI is a new method for assessing visceral fat distribution and dysfunction that can better predict the presence of diabetes, hypertension, and related cardiometabolism, as well as other more important fundamental aspects (31–33). The CMI is calculated by multiplying WHtR with TG/HDL-L. WHtR indicates the presence of fat in the body, and it has been found that abdominal obesity is strongly linked to infertility (34). TG/HDL-C is a strong predictor of metabolic syndrome and insulin resistance, playing a crucial role in the onset of infertility (35). The development of infertility and metabolic disorders is intricate and varied, with lipid metabolism disorders potentially playing a crucial role in follicular development, egg maturation, and hormone secretion (36). Women who follow a long-term high-fat diet may experience an accumulation of lipids in their eggs, leading to toxicity and hindering the maturation process (37). A large number of animal experiments have confirmed that dyslipidemia can lead to decreased female reproductive ability (38), and low-density lipoprotein receptor (LDLR) is a key factor in regulating lipid metabolism (39). Deletion of LDLR leads to dyslipidemia, which markedly decreases estrogen levels and fertility in female mice (40). Studies have shown that lipid metabolism disorders are associated with the reproductive performance of assisted reproductive technology, and hyperlipidemia is inversely correlated with pregnancy outcomes in patients with freeze-thaw embryo transfer (41). Other studies have shown that women with abnormal lipid metabolism have a higher risk of infertility (42). Hence, CMI has the ability to indicate the abnormal metabolic state within the body, allowing for the prediction of infertility risk and serving as a foundation for health promotion.

Our study benefits from a complex multi-stage probabilistic sampling design, which enhances the reliability and representativeness of our research. However, there are limitations to our study. Firstly, the cross-sectional analysis design prevents us from establishing a causal relationship between CMI and infertility. Additionally, due to database constraints, we were unable to include data on all covariates that may impact infertility and lipids in order to maintain a large enough sample size. Lastly, while we accounted for some confounders, we cannot completely eliminate the potential influence of other confounding factors. For example, polycystic ovary syndrome is a very common and important cause of infertility, but our study did not include people with PCOS, which may bias our findings. In addition, when performing sample exclusion, we only selected samples of women of childbearing age based on previous studies, which may have led to biased research results.

## 5 Conclusion

The prevalence of infertility in women in the United States is linked to CMI levels, with a higher CMI level corresponding to a higher prevalence of infertility. CMI has limited power in diagnosing infertility, and interestingly, the CMI-BMI index has good diagnostic power and can be used for early diagnosis of infertility. However, further prospective studies with large sample sizes are needed to confirm this relationship.

## Data Availability

The datasets presented in this study can be found in online repositories. The names of the repository/repositories and accession number(s) can be found in the article/supplementary material.
